# Illicit trade and real prices of cigarettes in Chile

**DOI:** 10.18332/tid/169785

**Published:** 2023-09-25

**Authors:** Guillermo Paraje, Luca Pruzzo, Mauricio Flores Muñoz

**Affiliations:** 1 Business School, Universidad Adolfo Ibáñez, Peñalolen, Chile; 2 Millennium Nucleus for the Evaluation and Analysis of Drug Policies (nDP), Santiago de Chile, Chile

**Keywords:** Chile, cigarettes, illicit trade

## Abstract

**INTRODUCTION:**

The tobacco industry claims that tobacco taxes are responsible for increased illicit trade in Chile, which they estimated at 37% in 2022. However, the evolution of cigarette consumption, estimated from population surveys, and of tax-paying cigarettes shows a decreasing penetration of illicit trade since 2018.

**METHODS:**

A gap analysis was used to estimate the evolution of illicit trade based on an arithmetic identity stating that total national cigarette consumption over a given period is equal to the registered consumption as paying taxes plus the cigarettes that are consumed nationally without paying taxes.

**RESULTS:**

Illicit trade penetration in Chile was around 10% in 2020, less than half of what the tobacco industry claimed. In addition, the evolution of real prices of cigarettes, calculated using tax collection data, indicates that real prices net of tobacco taxes increased significantly during 2015–2021, a period with no changes in tobacco taxation. The cheapest cigarettes, presumably competing with illicit cigarettes, registered the most significant price increase.

**CONCLUSIONS:**

Claims of increasing illicit trade penetration in Chile are unfounded and are not supported by data on consumption and tax-paying cigarettes.

## INTRODUCTION

The last tobacco tax increase in Chile was in 2014 when the tax structure was set to a specific component of approximately CLP 59.3 per stick (1000 Chilean Pesos about US$1.2), plus a VAT of 30% of the sales price, including taxes. Since then, the monopolistic tobacco firm, which domestically produces 98% of the cigarettes sold in Chile, started a persistent campaign in the media to associate this tax increase with an alleged rise in the illicit cigarette trade. Studies funded by this company and widely reproduced in mass media reported that illicit trade penetration went from 5.6% in 2014 to 22.3% in 2017 and 37.4% of the total market in 2022, increasing more than 16 percentage points since 2020^1^. An independent study in 2017 conducted in the Metropolitan Area of Santiago, Chile, found such a penetration to be 16.3%^2^.

This short report estimates the recent evolution in the illicit market in Chile through a gap analysis. In addition, it analyzes the behavior of average cigarette real prices over time to determine whether licit cigarette prices evolution is consistent with the tobacco industry’s (TI) alleged increase in illicit trade penetration.

## METHODS

The gap analysis estimates the evolution of illicit trade based on an arithmetic identity stating that total national cigarette consumption over a given period (*Q^T^*) is equal to the registered consumption as paying taxes (*Q^L^*), plus the cigarettes that are consumed nationally without paying taxes (*Q^I^*)^3^:

Q^T^=Q^L^+Q^I^.

Cigarettes that do not pay taxes are not necessarily illicit, as they can include cigarettes bought at duty-free shops or imported with legal personal customs franchises. Previous studies have shown that <1% of smokers buy cigarettes in duty-free shops or purchase them abroad using customs franchises^2^.

The registered consumption (*Q^L^*) is obtained from the Chilean Inland Revenue Service (SII for its Spanish acronym) statistics of cigarettes that pay national taxes. These statistics are not public and were obtained from the SII through a request under the Transparency Act.

Total cigarette consumption (*Q^T^*) is estimated from the National Drug Surveys in the General Population (ENPG) that were carried out in 2016, 2018 and 2020, which measure the number of smokers and consumption intensity among the population aged 12–65 years, irrespective whether that consumption is licit or illicit. These surveys recorded a monthly prevalence of tobacco consumption of 33.0%, 30.9%, and 28.4%, for 2016, 2018 and 2020, respectively (Supplementary file Table S1)^4^. Consumption for the in-between years was obtained by linear interpolation. Since they are survey-based estimates, 95% confidence intervals were calculated for these estimates.

Usually, quantities of *Q^T^* are under-reported^3,5^. An ‘uplift factor’ may account for under-reporting in such cases. This factor is calculated by dividing *Q^L^* and *Q^T^* in a particular year and used to upscale the estimates in other years (i.e. it is assumed that under-reporting is constant throughout time)^3,6^. Using such an uplift factor does not allow for estimating the penetration of illicit trade but just its trend over time. An alternative to such an ‘uplift factor’ that helps to assess the trend and the penetration of illicit trade is to assume a constant under-reporting level over time. Some authors have assumed different levels (e.g. 5%, 10%, 45%, etc.) and estimated illicit trade under these arbitrary scenarios^6,7^. In this study, we assume that the penetration of illicit trade was 15.2% in 2016, the level declared by the monopolistic tobacco firm^1^. This is not, in any way, an endorsement of such a figure but a way to consider a worst-case scenario for the estimation of levels and trends of illicit trade. As has been widely studied, TI estimates of illicit trade are systematically exaggerated and overestimate illicit trade figures^8^.

In Chile, tobacco firms set cigarette prices nationally and inform the SII, which publishes them. Using these published prices by brand, for the 2015– 2021 period, we computed weighted average prices by price quartiles, using as weights the brands’ market shares published by Euromonitor International^9^. Price quartiles (the cheapest is quartile 1, and the most expensive is quartile 4) are constructed the dividing the price range of all brands into four groups. With these real prices, an indicator of the net of taxes weighted real prices are calculated by price quartile. This is a proxy of the unitary price received by tobacco manufacturers. If illicit trade is increasing, it would be expected that tobacco firms operating in the licit market would be willing to maintain or even curtail real prices net of taxes for brands that directly compete with illicit brands (those at the lowest price quartile).

## RESULTS

**Figure 1:**
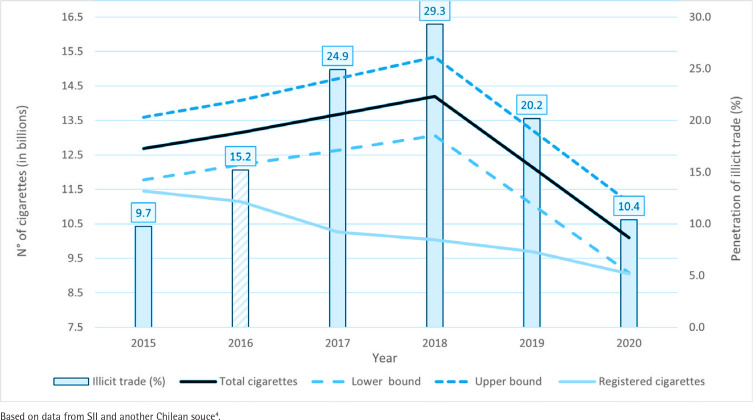
Estimated penetration of illicit trade in cigarettes in Chile, 2015–2020

Figure 1 shows the results of the gap analysis for Chile. During 2018–2020, there was a clear downward trend in the estimated total consumption of cigarettes. The fall in this consumption is greater than that of the tax-paying cigarettes, which implies a fall in the volume of non-tax-paying cigarettes. According to previous studies, most of this consumption is of illicit cigarettes^2^. The estimated penetration of illicit trade (plus the small fraction of legal non-tax-paying cigarettes) is 10.4% of the total market in 2020. Once again, it must be stressed that this estimate is ‘anchored’ to a likely initial overestimation of illicit trade in 2016. Even so, estimated levels of illicit trade for 2020 are low by international standards^10^. The tobacco firm, which has 98% of the local market, estimated that illicit trade in Chile in 2019 was 21.4% and for 2021 was 20.5%, at least double the estimate obtained in the present analysis^1^.

Regarding the evolution of the weighted real price net of taxes by price quartiles, Supplementary file Table S2 shows their annual percentage variation. The table shows, for instance, a 49% increase in such a real price for the cheapest quartile (and for the total of cigarettes) between 2015 and 2021. On the other hand, during 2020 and 2021, the cheapest quartile cigarettes increased by 24% and 6% in the real price net of taxes, respectively.

## DISCUSSION

The gap analysis showed that, at least until 2020, the illicit cigarette trade penetration in Chile had sharply decreased, reaching about 10% of the total market (including a small fraction of legal non-tax-paying cigarettes). This percentage was obtained considering the monopolistic tobacco firm’s likely overestimation of illicit trade penetration for 2016 as an initial estimate of illicit trade, which probably leads to overestimating the actual situation^2^.

The decline in the cigarette market’s size and penetration of illicit trade could be associated with the COVID-19 pandemic and the closure of the borders during much of 2020. Unfortunately, it is impossible to estimate the evolution in illicit trade for 2021 using the gap analysis because there are no estimates of cigarette consumption from population surveys. However, the consumption of legal tax-paying cigarettes showed a substantial increase during 2021 (20% relative to 2020, the first annual increase at least since 2014), which is also inconsistent with TI claims of higher illicit trade. More likely, the decrease in the illicit trade market since 2018 could be associated with the implementation in 2019 of a track-and-trace system that forced the real-time counting and marking of all tobacco legally sold in Chile. A recent study has shown that such implementation had an immediate result on fiscal revenues from tobacco taxes^11^. In the context of stable tobacco taxes, such an increase in fiscal revenues came from the increasing sales of tax-paying cigarettes referred to above.

In addition, the evolution in real cigarette prices shows that during 2020–2021, prices (especially for the cheapest cigarettes) increased substantially. Following the ‘economics logic’ of the TI, which associates an increase in the real price of legal cigarettes with an almost automatic increase in the size of the illicit market, one could argue that the price of legal cigarettes should not increase significantly in a context of intense competition from illicit cigarettes, especially those in the lowest price segment. In a market with relatively homogeneous products, where the possibilities of differentiation are severely limited due to the ban on advertising and where the discourse of the tobacco industry itself is that the products are close substitutes (otherwise, it would be untrue that increases in tobacco prices would almost automatically increase illicit trade), the entry of relatively cheaper illicit products in the market ought to imply a reduction in the prices of the cheap legal ones^12^. Some studies have documented the inconsistency in the tobacco industry’s argument that tax increases foster illicit trade, as this argument does not recognize that at least part of the price increase is directly attributable to the industry’s pricing strategies^13^.

Labor costs represent a relatively small part of total costs in a heavily capital-intensive activity like cigarette manufacturing^14^. These costs increased significantly less than the real sales prices in this period (12% vs 18%, respectively)^15^. The increase in average real prices of legal cigarettes could be due to the tobacco industry taking (even more significantly) advantage of the inelastic demand it faces to increase its profitability^16^. The absence of tobacco tax increases during 2015–2021 meant that the financial benefits of that higher average real price were captured by the tobacco industry instead of the public treasury (via higher tobacco taxes).

### Limitations

This study has some limitations. First, as usually happens with gap analyses, it is assumed that underreporting of cigarette consumption from users’ surveys (*Q^T^*) is constant over the years. There is no secondary information to determine if this assumption is realistic, though the fact that all surveys were collected over a relatively short period of time and using the same questionnaire and sampling methods can indicate that there is no reason not to take that assumption as valid. Second, gap analysis does not provide estimates of the actual market share of illicit cigarettes but the evolution of such a share over time. The estimate of the illicit market share is an arbitrary, worst-case scenario based on tobacco industry estimates, which tend to overestimate such a share. Thus, the estimates of illicit cigarette market shares obtained should not be considered as accurate but as a measure of what would be the case if such a worst-case scenario is materialized.

## CONCLUSIONS

Claims of increasing illicit trade penetration in Chile are unfounded and are not supported by data on consumption and tax-paying cigarettes. Contrary to tobacco industry claims and even using a worst-case scenario for illicit trade penetration, illicit trade is decreasing in Chile and is likely below average world levels. The evolution of real prices of legal cigarettes is also consistent with reduced pressures from the illicit market, as tobacco firms are increasing their unit sales revenues, especially for the cheapest cigarettes.

## Data Availability

The data supporting this research are available from the authors on reasonable request.
