# Supplementary material for: Illicit trade and real prices of cigarettes in Chile
Source: Tob Induc Dis. 2023 Sep 25;21:117. doi: 10.18332/tid/169785 (PMC10519129; doi:10.18332/tid/169785)
Supplement: Supplementary file 1 [file TID-21-117-s1.pdf]

Table S1. Estimated consumption from users' surveys and tax-paying cigarette data

Year	Total adult population (million) [1]	Smoking month-prevalence (%) [2]	Number of monthly smokers (millions) [3] = [1]*[2]	Average days smoked per month [4]	Average daily smoking intensity [5]	Self-reported annual consumption (billion sticks) [6]=[3]*[4]*[5]*12	Total annual consumption estimated (billion sticks) [7]	Annual tax-paid consumption (billion sticks) [8]	Number of illicit sticks (billion sticks) [9]=[7]-[8]	Illicit trade (% of total market) [10]=[9]/[7]
2015	10.08	33.8	3.41	21.3	8.0	6.98	12.68	11.45	1.23	9.7
2016	10.34	33.0	3.41	21.1	8.4	7.24	13.14	11.14	1.99	15.2
2017	10.66	32.0	3.41	20.9	8.8	7.53	13.67	10.26	3.40	24.9
2018	10.98	30.9	3.40	20.7	9.3	7.82	14.20	10.03	4.16	29.3
2019	11.06	29.7	3.28	21.4	7.9	6.69	12.15	9.69	2.45	20.2
2020	11.15	28.4	3.17	22.0	6.6	5.56	10.09	9.04	1.04	10.4

Total adult population, smoking prevalence, average daily smoking intensity, and self-reported consumption are estimated from the ENPG. The total consumption estimated is the self-reported consumption multiplied by the up-lifting factor. Annual tax-paid consumption is obtained from the SII. Column [7] is column [6] multiplied by the uplift factor.

Table S2: Annual percentage variation in real price (quartiles) net of taxes received by producers/distributors, 2015-2021

Real price net of taxes	2016	2017	2018	2019	2020	2021	Percentage change (2015-21)
Cigarettes Q1	-1	24	0	-4	24	6	49
Cigarettes Q2	-4	8	4	8	-5	-4	8
Cigarettes Q3	10	17	6	16	6	-7	47
Cigarettes Q4	9	14	14	13	0	-5	45
Weighted real average price net of taxes	8	24	4	2	13	0	49

Note: Q1 (cheapest), Q2, Q3 and Q4 (most expansive) represent the (unweighted) first, second, third, and fourth quartiles of the cigarette price distribution. The columns by year represent the percentage variation of that year compared to the previous ye ar, while the percentage change (2015-21) corresponds to the percentage variation of 2021 compared to 2015. The data used to construct this table were obtained from the SII.

© 2023 Paraje G. et al.
